# Physics-Informed Generative Adversarial Networks for Laser Speckle Noise Suppression

**DOI:** 10.3390/s25133842

**Published:** 2025-06-20

**Authors:** Xiangji Guo, Fei Xie, Tingkai Yang, Ming Ming, Tao Chen

**Affiliations:** Hangzhou Institute for Advanced Study, University of Chinese Academy of Sciences, Hangzhou 310024, China; guoxiangji18@mails.ucas.ac.cn (X.G.); xiefei24@mails.ucas.ac.cn (F.X.); yangtingkai@ucas.ac.cn (T.Y.); chentao@ucas.ac.cn (T.C.)

**Keywords:** laser illumination, speckle noise, physics-informed, noise suppression

## Abstract

In high-resolution microscopic imaging, using shorter-wavelength ultraviolet (UV) lasers as illumination sources is a common approach. However, the high spatial coherence of such lasers, combined with the surface roughness of the sample, often introduces disturbances in the received optical field, resulting in strong speckle noise. This paper presents a novel speckle noise suppression method specifically designed for coherent laser-based microscopic imaging. The proposed approach integrates statistical physical modeling and image gradient discrepancy into the training of a Cycle Generative Adversarial Network (CycleGAN), capturing the perturbation mechanism of speckle noise in the optical field. By incorporating these physical constraints, the method effectively enhances the model’s ability to suppress speckle noise without requiring annotated clean data. Experimental results under high-resolution laser microscopy settings demonstrate that the introduced constraints successfully guide network training and significantly outperform traditional filtering methods and unsupervised CNNs in both denoising performance and training efficiency. While this work focuses on microscopic imaging, the underlying framework offers potential extensibility to other laser-based imaging modalities with coherent noise characteristics.

## 1. Introduction

In high-precision microscopic imaging, ultraviolet (UV) lasers are widely used as illumination sources to overcome the resolution limits imposed by optical diffraction [1,2]. Although broadband light sources can alleviate interference and wavelength-dependent limitations in many applications, their widespread use is restricted by high costs and aberrations caused by chromatic dispersion. In contrast, single-wavelength lasers provide a cost-effective alternative due to their low cost, high power output, and ability to reduce spectral interference. However, the high coherence inherent to single-wavelength lasers inevitably leads to speckle noise during sample observation, which results from phase interference effects in the imaging process. This phenomenon significantly degrades image quality and presents substantial challenges for subsequent analytical processing.

Speckle noise is a common and often detrimental artifact in many laser-based imaging systems due to the coherent nature of the light source. Various imaging modalities—such as laser scanning microscopy [3], confocal microscopy [4], digital holography [5,6], infrared absorption imaging [7], and laser speckle contrast imaging (LSCI) [8]—suffer from speckle artifacts that degrade spatial resolution and obscure structural details.

The spatial distribution, size, and contrast of speckle patterns vary depending on imaging geometry, laser wavelength, optical components, and sample properties. In recent years, extensive research has been conducted on speckle suppression by numerous scholars [3,4,9,10,11], with the research primarily progressing along two major directions: optical hardware solutions and software algorithmic approaches [6,12,13,14].

On the hardware side, traditional rotating phase plates and ground glass diffusers are commonly used as decoherence elements, but they often significantly reduce illumination energy. Electrically actuated micro-optical diffusers have been employed to modulate laser coherence [15], effectively reducing speckle noise while improving imaging uniformity and accuracy. However, their application is limited by high equipment costs and complex integration processes. Another effective hardware-based technique involves optimizing the phase of a spatial light modulator (SLM) and using a rotating phase mask [16]. This method suppresses speckle noise without compromising image contrast, but its performance is highly sensitive to exposure time, posing certain challenges in practical applications. Fridman et al. [17] proposed a speckle reduction technique based on the variation in pinhole position, achieving a 36% suppression of speckle contrast and causing only minor imaging loss. This technique holds significant value in clinical examinations using reflectance confocal microscopy. Additionally, Xu et al. [18] proposed a thermal lens compensation method to reduce spatial coherence, which outperforms Gaussian beams in atmospheric turbulence imaging. However, its reliance on a precise thermal control system limits its scalability and general applicability.

Removing speckle noise through optical hardware often requires the addition of extra decoherence elements in either the temporal or spatial domain, which can reduce illumination brightness. Therefore, backend processing using algorithmic approaches is equally important. Bianco et al. [5] described various speckle suppression strategies in digital holographic scenarios, among which the algorithm highlighted the effectiveness of repetitive image acquisition combined with traditional denoising methods in speckle suppression. However, in many practical cases, it is impossible to obtain multiple repetitive area images. So traditional denoising algorithms, such as median filtering, Wiener filtering, and block-matching 3D filtering (BM3D) [19], have played an important role in speckle noise suppression and early-stage image restoration.

Various enhanced BM3D variants have been proposed for different imaging modalities, including hyperspectral, terahertz, and holographic imaging [20,21,22,23,24], and may offer improved performance if adapted to the coherent speckle context. Among these, spatial displacement-based reconstruction methods can reduce speckle noise while preserving resolution; however, these methods rely on high-quality image acquisition and are not suitable for time-sensitive applications [25]. Several extensions such as the SPAR (Sparse Phase and Amplitude Representation) framework developed by Katkovnik et al. [26] have shown excellent performance in suppressing speckle noise under coherent illumination by modeling both phase and amplitude in the complex domain.

Another approach is non-local filtering combined with Pearson correlation and a Butterworth high-pass filter (NLM-PB), which can effectively suppress noise while preserving details, but its performance degrades in complex backgrounds [27]. Wavelet shrinkage methods [28] offer multi-scale decomposition capabilities, yet often require carefully tuned thresholds and may suppress fine structural features. Total variation (TV) regularization [29] is edge-preserving but tends to oversmooth the repetitive microstructures common in semiconductor and microscopy images. Anisotropic diffusion filtering [30], though effective in some speckle scenarios, is highly sensitive to the selection of edge-stopping functions and noise levels.

Compared with these methods, deep learning models, once trained, offer faster inference and greater flexibility, though at the cost of reduced interpretability and dependence on training data. Deep learning methods, such as multi-scale convolutional neural networks (CNNs), offer fast denoising performance but exhibit weak generalization across different noise patterns [31]. Chen et al. [32] simulated speckle noise by combining Gaussian and speckle noise and trained a Swin Transformer-based model, which showed promising results on simulated data but exhibited limited generalization capability. Furthermore, CNN-LSTM-based speckle spectrometers have demonstrated improved speed and accuracy in spectral reconstruction; however, their training requires paired images, leading to high data acquisition costs and increased implementation complexity [33]. More recently, unsupervised and self-supervised deep learning methods have emerged as promising alternatives. Noise2Void [34] and Noise2Self [35] learn denoising models from single noisy images by masking pixels during training, assuming noise independence. However, these assumptions do not hold for spatially correlated speckle, limiting their performance. There are also domain-specific methods such as Liu et al.’s self-supervised speckle suppression for OCT [36]. While these methods have made significant contributions, their effectiveness is often limited by the lack of physical interpretability and their reliance on assumptions that break down in highly structured or physically driven noise fields. This motivates the development of physics-informed denoising frameworks that integrate optical knowledge into the learning process.

In many scenarios, although we cannot obtain completely paired images, it is easy to obtain two sets of data with different styles. At this time, a Cycle Generative Adversarial Network (CycleGAN) [37] is an extremely effective method. However, the loss of this method itself may cause image artifacts after training, and this often requires further constraints. In recent years, physics-informed deep learning methods have gained significant attention across various fields. Raissi et al. [38] proposed the concept of physics-informed neural networks (PINNs), which can effectively guide network training in scenarios governed by physical constraints and partial differential equations (PDEs). Beyond fields such as fluid dynamics and PDE-based computations, researchers have also developed PINN-based algorithms for image super-resolution [39] and image denoising [40], achieving remarkable results. Incorporating physical laws into deep learning training is becoming a major trend in physics-informed deep learning research, as it not only directs the network toward an optimal solution more efficiently but also typically reduces the required training data.

In this paper, the physical properties of speckle patterns in laser imaging are analyzed to design a speckle-related physical constraint and gradient constraint, which are integrated into a CycleGAN. The proposed method achieves effective speckle noise suppression under small datasets. The structure of this paper is as follows: Section 1 reviews the current state of research and the significance of this study. Section 2 provides a detailed explanation of the proposed method. Section 3 presents experimental results to validate the effectiveness of the proposed approach. Finally, Section 4 summarizes the contributions and findings of this work. We focus specifically on coherent laser-based microscopic imaging in this study, where speckle noise severely degrades the visualization of fine structures due to high spatial coherence and surface micro-roughness. To clarify, while we do not explicitly model how hardware parameters such as aperture size or wavelength affect speckle size, our method captures general speckle-induced perturbations in a system-agnostic way through statistical and structural constraints.

## 2. Method

### 2.1. Speckle Phenomenon Analysis

In laser-based imaging systems, the use of high spatial coherence illumination inevitably introduces speckle noise. This phenomenon originates from the interference of coherent waves reflected from rough or partially rough surfaces, leading to phase fluctuations and irregular amplitude modulations across the imaging plane. The resulting complex optical field $E_{s}(x,y)$ can be described as follows:

(1) $$E_{s}(x,y)=E_{0}(x,y)\cdot e^{i\phi (x,y)}$$

where $E_{0}(x,y)$ is the ideal, speckle-free field, and ^*ϕ*(*x*,*y*)^ is the random phase induced by surface roughness and system scattering. The recorded intensity becomes the following:

(2) $$I(x,y)={\left|E_{s}(x,y)\right|}^{2}={\left|E_{0}(x,y)\right|}^{2}\cdot {\left|e^{i\phi (x,y)}\right|}^{2}={\left|E_{0}(x,y)\right|}^{2}$$

In fully developed speckle fields, where the surface is entirely diffuse, the intensity statistically follows a negative exponential distribution [41]:

(3) $$p(I)=\frac{1}{2\sigma^{2}}\exp(-\frac{I}{2\sigma^{2}})$$

where σ is the standard deviation of the random speckle background. However, in most practical imaging scenarios, surface roughness is partial, and both coherent and incoherent reflections contribute to the measured signal. Under this condition, the intensity follows the Rician distribution [42]:

(4) $$p(I)=\frac{1}{2\sigma^{2}}\exp\left(-(\frac{I+A^{2}}{2\sigma^{2}})\right)I_{0}(\frac{A\sqrt{I}}{\sigma^{2}})$$

Here, A is the coherent amplitude, and $I_{0}(x)$ is the zeroth-order modified Bessel function of the first kind. The value of A/σ determines the statistical nature of the speckle pattern, from exponential-like to Gaussian-like. Since the imaged sample includes high-reflectivity aluminum patterns on a transparent substrate in this study, the observed speckle often exhibits non-zero mean characteristics. Therefore, Rician distribution is used to better capture the noise statistics in these regions.

In addition to intensity perturbation, speckle noise introduces significant fluctuations in the image gradient field. Applying the chain rule to Equation (1), the image gradient can be expressed as follows:

(5) ∂I∂x=2Re(Es*(∂E0∂x+iE0∂ϕ∂x)eiϕ)

This equation shows that speckle alters both the intensity and the structure of the image, particularly in the high-frequency gradient domain. The actual image gradient field can therefore be approximated as follows:

(6) $$\nabla I(x,y)\approx \nabla I_{0}(x,y)+N(x,y)$$

where N(x,y) denotes the gradient fluctuation due to speckle interference.

**Figure 1:**
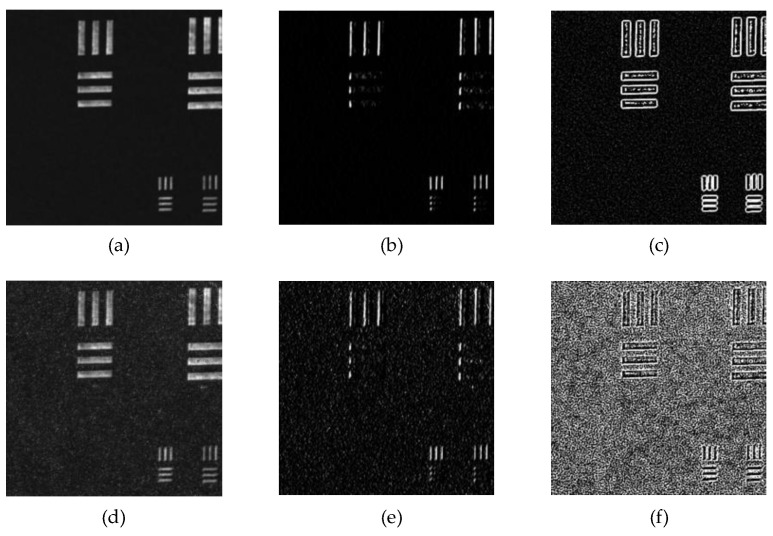
Gradients and divergence of the image before and after speckle influence. (**a**–**c**) represent the clean (speckle-free) original image, the x-direction gradient map, and the divergence map, and (**d**–**f**) represent the speckle-affected original image, the x-direction gradient map, and the divergence map, respectively.

Figure 1 illustrates the contrast of gradients and divergences in the x-direction before and after the influence of speckle noise. The gradient and divergence represent the trends in the image, thereby amplifying the impact caused by speckle noise. Compared to the imaging itself, the gradient and divergence are more significantly affected by speckle noise. After light field modulation by speckle noise, the image’s grayscale statistics and details undergo considerable changes. This aspect pertains to the alteration of the image due to physical information. In the use of deep learning for speckle noise suppression, analyzing relevant data from the image can effectively guide the network’s training process.

**Figure 2:**
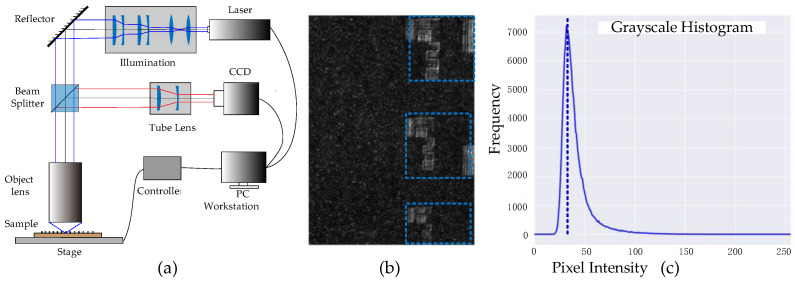
(**a**) Schematic diagram of the imaging system studied in this paper. (**b**) Micrograph acquired by the system; the area within the blue box is the pattern texture region, and the rest is the background region. (**c**) Histogram statistics of the image, which effectively reflect the statistical characteristics of the speckle field, the dashed line serves as a visual reference for the main intensity peak in the grayscale histogram.

This paper is based on the optical system shown in Figure 2a. The system uses coaxial laser illumination, where the illumination optical path is fully integrated with the laser source and precisely aligned as an independent subsystem. The system achieves an 80× magnification, uses a beamsplitter with a 1:1 splitting ratio, and is equipped with an ultraviolet-enhanced CCD camera as the detector. Since the system employs a UV-transmissive objective lens, a 355 nm laser is selected as the illumination source. After passing through the illumination optics, the beam is expanded into a uniform spot with a diameter of 30 mm. In addition to the coherent 355 nm laser, the imaging system also employs a broadband plasma laser covering the spectral range of 260–450 nm. This source exhibits low spatial coherence, which effectively eliminates laser-induced speckle noise. It shares the same optical path as the 355 nm laser via a switchable beam combiner. The broadband illumination enables the acquisition of reference images that are free from coherent interference, providing a suitable target domain for unsupervised training. After precise adjustment, the system is capable of high-resolution microscopic imaging. Figure 2b,c show the spatial image and its corresponding grayscale histogram. It can be observed that the histogram statistics satisfy the light intensity distribution function of partial scattering.

In our imaging setup, the speckle pattern arises primarily due to the high spatial coherence of the 355 nm laser and the partial roughness of the aluminum-on-glass substrate. The imaging configuration, including the fixed aperture of the UV objective and the coaxial illumination design, produces speckle fields with moderate size and contrast. Although we do not vary the system parameters in this study, the proposed method remains effective across different sample regions exhibiting variable speckle characteristics, as shown in our experiments.

### 2.2. Network Architecture

When obtaining the dataset, although it is difficult to obtain paired images with both speckle noise and no speckle noise for deep ultraviolet microscopic imaging, it is relatively simple to obtain a group of images without speckle noise using a small-power broadband plasma laser. Therefore, by conducting style learning on two unpaired images, we can achieve good noise suppression, and the CycleGAN is the most suitable basic method in this scenario. CycleGAN [43] is an unsupervised deep learning model used for image style transfer. Unlike traditional deep learning models [37] that rely on fully labeled paired datasets, CycleGAN performs denoising learning without paired data by imposing constraints on both the generator and discriminator during training. This method achieves effective noise suppression through cyclic learning between two distinct data domains. We assume that the image p with speckle noise follows the distribution $p\sim P_{\mathrm{noisy}\;}(p)$, and the clean image q follow distribution $q\sim P_{\mathrm{clean}\;}(q)$. The generator G is responsible for converting the noisy image into a clean image, while the generator F is responsible for converting the clean image into a noisy image.

**Figure 3:**
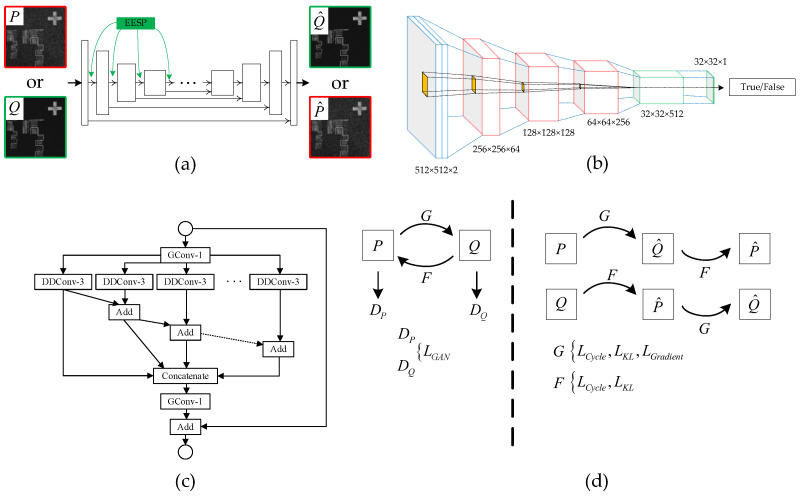
(**a**) Schematic diagram of the generator, where G and F share the same network architecture. (**b**) Network structure diagram of the discriminator. The red module represents the downsampling operation, while the green module indicates the operation without downsampling. (**c**) The EESP module used in the generator. DDConv refers to depth-wise dilated convolution, and GConv refers to group pointwise convolution. (**d**) Description of the specific application of the loss function during the network training process.

As shown in Figure 3a, the basic building block of the generator in this paper is chosen to be an encoder–decoder architecture, specifically the U-shaped module. Similar to the structure of U-Net, the U-shaped module is a fully convolutional encoder–decoder structure. In the encoding part, this paper introduces an extremely efficient spatial pyramid (EESP) [44] to increase the receptive field with minimal parameters, enhancing the network’s performance. The structure of EESP is shown in Figure 3c, the EESP module architecture consists of a group of parallel depth-wise dilated convolutions (DDConv-3) with varying dilation rates, enabling multi-scale receptive field aggregation. The input first passes through a group pointwise convolution (GConv-1) for channel compression. The compressed features are then processed in parallel by several DDConv-3 blocks. Intermediate results are hierarchically aggregated through element-wise addition, and all outputs are concatenated to form a multi-scale representation. The concatenated tensor is passed through another GConv-1 layer to fuse the features. Finally, a residual connection is added between the module input and output to preserve the original feature information and facilitate gradient flow.

The encoding path maps the input domain’s image to an abstract representation using four EESP blocks, with each convolutional layer followed by batch normalization (BN) and a Leaky-ReLU [45] activation function. Within the U-shaped module, the corresponding feature maps from the encoder and decoder are directly concatenated and then continue with upsampling. This ensures that low-level feature maps are effectively utilized, preventing the loss of image features during the downsampling process at the encoder–decoder bottleneck. In the encoding path, pooling is not used; instead, downsampling is achieved by using convolution with a stride of 2 at the last convolution of the EESP block. Except for the final convolution layer, which uses Tanh [46] as the activation function, all convolution layers use ReLU as the activation function. In the decoding path, the feature maps come not only from the decoder’s bottom-up feature maps but also from the encoder part.

To achieve better discrimination performance, this paper employs PatchGAN as the discriminator. As shown in Figure 3b, the PatchGAN discriminator architecture takes a 512 × 512 × 2 input (blue block), representing the concatenation of a real and a generated image. It passes through a series of convolutional layers (red blocks), where the spatial resolution is progressively reduced and the number of feature channels is increased, resulting in feature maps of sizes 256 × 256 × 64, 128 × 128 × 128, 64 × 64 × 256, and finally 32 × 32 × 512. This is followed by a set of convolutional layers without further downsampling (green blocks), which refine deep feature representations. A final 1 × 1 convolution outputs a 32 × 32 × 1 probability map, where each value corresponds to the authenticity prediction of a local image patch. Yellow blocks in the diagram indicate convolutional filters. The input to the discriminator consists of the noisy image and the denoised image concatenated together. After each convolution operation, batch normalization and ReLU activation layers are applied. The discriminator improves the generator’s inference performance in a more detailed manner by evaluating each point within the patches.

### 2.3. Physics-Informed Loss Function

The traditional CycleGAN loss function consists of two components: the adversarial loss $L_{GAN}$ and the cycle consistency loss $L_{\mathrm{cycle}\;}$, given by the following:

(7) $$\begin{array}{c} L_{GAN}=E_{q∼P_{clean}(q)}[\log D_{clean}(q)]+E_{p∼P_{noisy}(p)}[\log(1-D_{clean}(G(p)))]\\ \;\;+E_{p∼P_{noisy}(p)}[\log D_{noisy}(p)]+E_{q∼P_{clean}(q)}[\log(1-D_{noisy}(F(q)))] \end{array}$$

(8) $$L_{\mathrm{cycle}\;}=E_{p\sim P_{noisy}(p)}[{\left\|F(G(p))-p\right\|}_{1}]+E_{q\sim P_{clean\;}(q)}[{\left\|G(F(q))-q\right\|}_{1}]$$

Adversarial loss encourages the generators G and F to produce outputs that are indistinguishable from real images by their respective discriminators. Specifically, $D_{clean}$ distinguishes real clean images from those generated by G while $D_{noisy}$ distinguishes real noisy images from those generated by F. The cycle-consistency loss ensures that the mappings are invertible by minimizing the reconstruction error between the original image and the image obtained after a forward and backward mapping. This combination enables unpaired image translation while preserving the underlying structure of the images. The two loss functions mentioned above impose constraints on the entire network’s inference process, but in practical applications, they may produce artifacts, thus reducing the denoising performance. To effectively suppress speckle noise under unpaired training conditions, we introduce two physics-informed loss functions that do not rely on pixel-level supervision or background masks. These losses are derived from the statistical and structural characteristics of laser-induced speckle noise and guide the generator toward producing visually clean and physically consistent images. Specifically, we propose a distribution-level KL divergence loss and a structure-aware gradient consistency loss.

Laser speckle, arising from multipath interference under coherent illumination, fundamentally alters the statistical distribution of image intensity. In background regions, the intensities tend to follow a negative exponential distribution, while partially structured areas often exhibit Rician characteristics. In contrast, target domain images acquired under incoherent or low-coherence conditions exhibit smoother and more concentrated intensity distributions. To constrain the generator to mimic these statistical properties, we define a KL divergence loss between the grayscale histogram of the generated image and that of the target domain:

(9) $$L_{KL}=\sum_{i}P_{G}(I_{i})\log(\frac{P_{G}(I_{i})}{P_{Y}(I_{i})})$$

where $P_{G}(I_{i})$ and $P_{Y}(I_{i})$ denote the intensity distributions of the generated and target domain images, respectively. The target distribution is precomputed from a large number of real target domain images, while the generated distribution is estimated dynamically from each training batch using a smoothed histogram, which helps reduce the impact of sparse bin counts and ensures stable KL divergence computation during training. This loss encourages the generator to eliminate speckle-induced heavy tails and conform to the global statistical behavior of clean images.

In addition to intensity deviation, speckle noise introduces high-frequency fluctuations in the gradient field, especially along object boundaries. However, in the absence of ground-truth images or annotated masks, it is not feasible to directly supervise gradients. To address this, we propose a self-guided gradient consistency loss based on a pseudo-clean reference image. The input speckled image is first passed through a Gaussian filter to obtain a smoothed version that suppresses high-frequency noise while preserving structural content. The gradients of this smoothed image are then used as a weak supervisory signal to guide the restoration of structural features. The loss is defined as follows:

(10) $$L_{grad}=\frac{1}{\left|\Omega \right|}\sum_{\left(x,y\right)}\left|\nabla G(x)-\nabla \tilde{x}\right|$$

where $\tilde{x}=GaussianBlur(x)$, and ∇ denotes the spatial gradient operator (e.g., Sobel). This formulation allows the generator to retain sharp edges and suppress phase-induced oscillations without requiring explicit supervision. Finally, the total objective for the generator integrates adversarial loss, cycle-consistency loss, and the proposed physical constraints as follows:

(11) $$L_{total}=L_{GAN}+L_{cycle}+\lambda_{1}L_{KL}+\lambda_{2}L_{grad}$$

where $\lambda_{1}$ and $\lambda_{2}$ are hyperparameters that balance the statistical and structural constraints. These two physics-informed losses form a lightweight yet effective supervisory signal that enables the model to recover clean, edge-preserving representations from heavily speckled laser images in an entirely unsupervised manner. The loss function does not always play a role during the training process. As shown in Figure 3d, the gradient loss is only used as a constraint when generating the denoised image from the noisy image.

### 2.4. Dataset and Training Method

This study adopts an unsupervised learning approach, where the training dataset consists of images from two different domains, thus eliminating the need for paired samples. In this research, images are collected using a 355 nm laser and 260–450 nm broadband plasma lasers, as described in the optical system in Section 2.1. The observed sample is an electron beam lithography sample, using a glass substrate with metal aluminum patterns lithographed onto it. The background appears black due to light transmission, while the pattern reflects light and appears white. After image acquisition and simple augmentation (including horizontal flipping, slight rotation, and brightness adjustment), each domain of the training set contains 5000 grayscale images. These images are not captured from a single static region but from multiple areas across the same aluminum-on-glass lithographic sample. During acquisition, the sample stage was incrementally shifted to capture different microstructures across the patterned substrate. In addition, due to the high spatial coherence of the 355 nm laser source, even small variations in sample position, orientation, or ambient conditions cause noticeable changes in the speckle pattern. This results in frame-to-frame speckle decorrelation, allowing the network to observe diverse speckle realizations during training and improving the model’s generalization capability. The test dataset includes 500 pairs of independently collected images, each consisting of a speckle-contaminated image captured under coherent 355 nm laser illumination and a corresponding speckle-free image acquired using a broadband plasma laser (260–450 nm). Due to the lower energy output of the plasma laser, the exposure time for speckle-free imaging was increased fourfold to enhance contrast and ensure the capture of fine structural details. To enable quantitative evaluation, all image pairs were acquired from the same regions and aligned using image registration techniques to ensure pixel-wise correspondence.

We used the Adam optimizer [47], which is an adaptive gradient-based optimization algorithm that combines momentum and adaptive learning rate estimation, to accelerate convergence during training, with hyperparameters for the two moment estimates set to 0.9 and 0.999 and the exponential decay rate for the first moment estimate set to 0.5. The initial learning rate was set to 0.01. Given the resolution of the network input, the batch size is set to 8 during training. The generator and discriminator are trained alternately for a total of 200 epochs.

## 3. Experiments

### 3.1. Evaluation Metrics

In order to effectively evaluate the speckle noise suppression effect, this paper selects multiple evaluation metrics for result analysis. First, the speckle contrast, which intuitively assesses the impact of the speckle field, is defined as follows:

(12) $$C\;=\frac{\sigma }{\mu }$$

where σ and μ are the mean and variance of the image after noise suppression, respectively. Additionally, this paper introduces the signal-to-noise ratio (SNR) as an evaluation metric for local effects. The SNR is defined as follows:

(13) $$\mathrm{SNR}=\frac{\mu_{t}-\mu_{B}}{\sigma_{B}}$$

In the equation, $\mu_{t}$ is the mean of the target region, $\mu_{B}$ is the mean of the background region near the target, and $\sigma_{B}$ is the standard deviation of the background region near the target. The SNR of the denoised image in the local region can effectively evaluate the performance of the algorithm.

To better evaluate the difference between the network inference results and the images with truly no coherent light illumination-induced speckle field disturbance, this paper uses mean squared error (MSE) and structural similarity index measure (SSIM) as evaluation metrics. The definitions of MSE and SSIM are as follows:

(14) $$\mathrm{MSE}=\frac{1}{m\times n}\sum_{i=0}^{m-1}\sum_{j=0}^{n-1}{\left(I_{0}(i,j)-I(i,j)\right)}^{2}$$

(15) $$\mathrm{SSIM}=\frac{\left(2\mu_{x}\mu_{\hat{x}}+c_{1})(2\sigma_{x\hat{x}}+c_{2}\right)}{\left(\mu_{x}^{2}+\mu_{\hat{x}}^{2}+c_{1})(\sigma_{x}^{2}+\sigma_{\hat{x}}^{2}+c_{2}\right)}$$

where *m* and *n* are the resolution of the images, which are both 512 in this study, and $I_{0}$ and I represent the reference image without speckle and the speckle-suppressed image, respectively. MSE represents the difference between the two images, with smaller values indicating better correction effects. On the other hand, SSIM represents the similarity between the two images, with higher values indicating better correction effects. $\mu_{\hat{x}}$ and $\mu_{x}$ are the mean values of the generated image $\hat{x}$ and the reference image x, and $\sigma_{\hat{x}}^{2}$ and $\sigma_{x}^{2}$ are the variances of $\hat{x}$ and x. $\sigma_{x\hat{x}}$ is the covariance between $\hat{x}$ and x, and $c_{1}$ and $c_{2}$ are constants used to avoid division by zero. In this paper, $c_{1}$ and $c_{2}$ are both set to 0.001.

### 3.2. Ablation Experiment

In this study, we optimized the network structure and loss function. To investigate the effectiveness of the proposed method, we conducted ablation experiments to analyze the contributions of the improved network and the physical information to the overall performance of the algorithm.

#### 3.2.1. Network Structure

Compared to the traditional CycleGAN network structure, the method proposed in this paper mainly focuses on improving the generator structure. To explore the impact of different structures on network performance, we trained different generator architectures using the same training set and tested them on the same dataset. The generator structures used in the experiments are the following:

1. Simple encoder–decoder structure;
2. Standard U-Net network structure;
3. U-Net structure with additional EESP blocks (U-Net + EESP). The discriminator and network training strategy were kept consistent to avoid the influence of other factors on the results.

The results of the ablation experiment are shown in Table 1. Different generator network structures exhibit varying levels of performance. Compared to the simple encoder–decoder structure, U-Net shows significant improvement, mainly due to the skip connections, which utilize shallow features to enhance network performance. In the U-Net + EESP group, experiments demonstrate that the EESP structure benefits the U-Net module in extracting speckle field information. The increased receptive field enables the network to focus more on contextual information.

#### 3.2.2. Loss Function

To evaluate the impact of the loss function on network training, we analyzed its effect under different weights. Since adversarial loss and cycle consistency loss are conventional losses, the use of these two losses is set as the baseline. By adjusting the weights of the three proposed losses, we examined the guidance provided by physical information during network training. In the evaluation of individual loss functions, the weights of other losses are fixed at 1, and the weight of the target loss is varied from 0, 0.1, 0.2, 0.5, 0.8, 1, 2, to 5 for experimental conditions. To assess the training effect, we selected the adversarial loss, cycle consistency loss, and overall loss as the dependent variables for analysis.

(a) KL Loss

**Figure 4:**
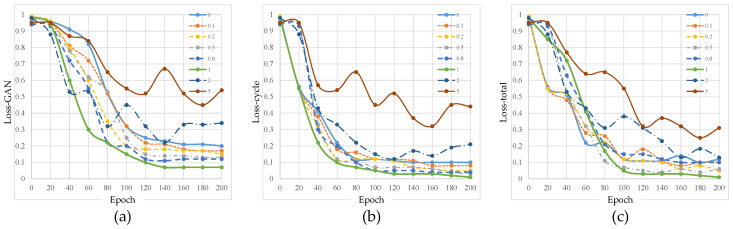
Training curves under different KL loss weights. (**a**–**c**) represent the curves of adversarial loss, cycle consistency loss, and total loss as a function of training epochs, respectively. Data for the figure are selected every 20 epochs.

Figure 4 shows the training effects under different KL loss weights. When the KL coefficient is small (0~0.2), its influence is weak, and the other losses dominate the training, causing the curve to steadily decline. When the KL loss coefficient is moderate (0.5~1), the KL constraint starts to take effect, and the loss decreases rapidly without interfering with GAN training, resulting in the best overall training effect. However, when the KL loss coefficient is too large (2–5), the KL constraint becomes too strong, causing the adversarial loss curve to oscillate and rise. This suggests that the model may over-constrain the distribution matching, negatively affecting the generation quality. Therefore, the optimal KL loss weight is between 0.8 and 1, balancing distribution matching and adversarial training effects.

Table 2 shows the final quantitative results under different KL loss weights. In the task of denoising speckle noise in images, the choice of KL loss weight directly affects the denoising performance. When the weight is low (0~0.5), the denoising ability is weak, the speckle contrast is high, and although the SSIM is relatively high, the MSE remains large, resulting in incomplete denoising. When the weight is moderate (0.5~2), the denoising effect is the best, the speckle contrast is significantly reduced, the SNR increases, and both SSIM is highest and MSE is lowest, effectively balancing denoising with image detail preservation. However, when the weight is too high, although the speckle suppression is stronger, the image becomes overly smooth, causing loss of details, a decrease in SSIM, and an increase in MSE. Therefore, the KL loss weight should be controlled between 0.8 and 1 to achieve optimal denoising performance and image quality.

(b) Gradient Loss

**Figure 5:**
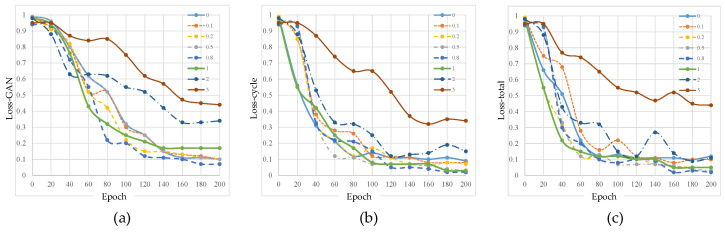
Training curves under different gradient loss weights. (**a**–**c**) represent the curves of adversarial loss, cycle consistency loss, and total loss as a function of training epochs, respectively.

The training curve for different gradient loss weights is shown in Figure 5. The change trend of the loss curve is as follows: when the gradient loss coefficient is small (0~0.2), the impact on the training is minimal, and the cycle consistency loss decreases quickly, allowing the model to maintain consistency during domain translation. A smaller gradient loss has little overall impact on the training, and the denoising effect is weaker. When the gradient loss coefficient is moderate (0.5~1), the adversarial loss remains relatively stable, but the gradient constraint strengthens, which affects the adversarial generation to some extent. The cycle consistency loss still shows a good decreasing trend, and the image translation quality remains good. As the gradient loss gradually decreases, the model successfully matches the target distribution during the denoising process, and the denoising effect is significantly enhanced. When the gradient loss coefficient is large (5~10), the adversarial loss increases significantly, indicating that the generator is under stronger constraints, causing the generated image to become blurry or lose structure. The cycle consistency loss decreases more slowly or even oscillates, which suggests that the model may struggle to balance denoising with maintaining the original structure. The gradient loss initially decreases rapidly but may oscillate in the later stages, suggesting that the model might overfit the gradient constraint, which affects the overall training stability.

As it shows in Table 3, when the gradient loss coefficient is small (0~0.2), the denoising ability is weak, and the model struggles to match the gradient distribution. The MSE and SSIM values are mediocre, but the results gradually improve as the loss function guides the network. At a weight of around 0.8, all four evaluation metrics achieve optimal values. However, when the weight increases further, the network training becomes unstable, and the quantitative metrics fail to achieve ideal results. Therefore, the gradient loss weight should be between 0.5 and 1 to achieve optimal training for the network.

Finally, we conducted ablation experiments by setting the weights of KL loss and gradient loss to 1 and 0.8, respectively. Adversarial loss and cycle consistency loss are set to 1 by default. The ablation experiment results for the different loss functions are shown in Table 4. All loss functions can train the network to some extent, but the KL loss function performs the best when trained alone. The combined loss effect of KL and gradient losses is the best when these two losses are fused.

### 3.3. Dataset Scale Experiment

In order to verify the impact of the proposed physical information on training and results with different dataset sizes, we conducted experiments by varying the size of the training set. We trained the model using 1000, 2000, and 5000 images in the training set to investigate the effect of dataset size on denoising performance, and we observed the final speckle suppression results. The primary comparison was made between the baseline method where $\lambda_{1}$, $\lambda_{2}$, and $\lambda_{3}$ are set to 0, and the proposed loss function with weight ratios of 1, 0.8, and 2 are for $\lambda_{1}$, $\lambda_{2}$, and $\lambda_{3}$.

**Figure 6:**
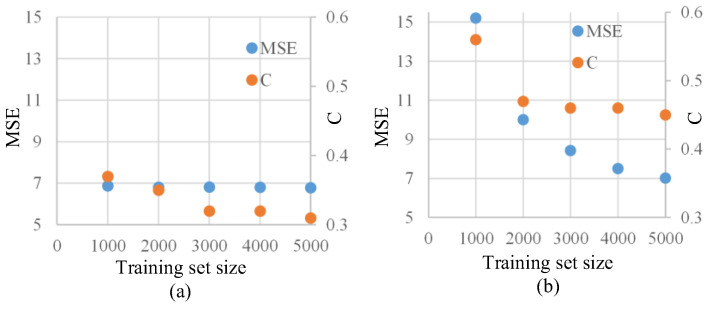
Results of different training set sizes under two loss functions. (**a**) Scatter plot of the results using the proposed physics-informed neural network. (**b**) Results using the default loss function.

The effect of changing the dataset size on speckle suppression is shown in Figure 6, where MSE and speckle contrast (C) are selected as evaluation criteria. The results indicate that under the default loss function, the network’s training requires a large dataset, and as the dataset size decreases, the training performance noticeably deteriorates, making convergence difficult. However, by incorporating the proposed joint physical information constraint, the network can avoid underfitting even with reduced training data, achieving acceptable results despite the data shortage.

### 3.4. Comparison Experiment

We tested the model using 500 noisy images containing different observation sample areas and detector settings. Since the proposed method does not require paired datasets, its performance was compared with several classic digital image denoising methods and the latest unsupervised deep learning methods [48,49]. Although multi-image-based noise suppression algorithms are commonly used for image enhancement, this study focuses only on the single-image input scenario.

**Figure 7:**
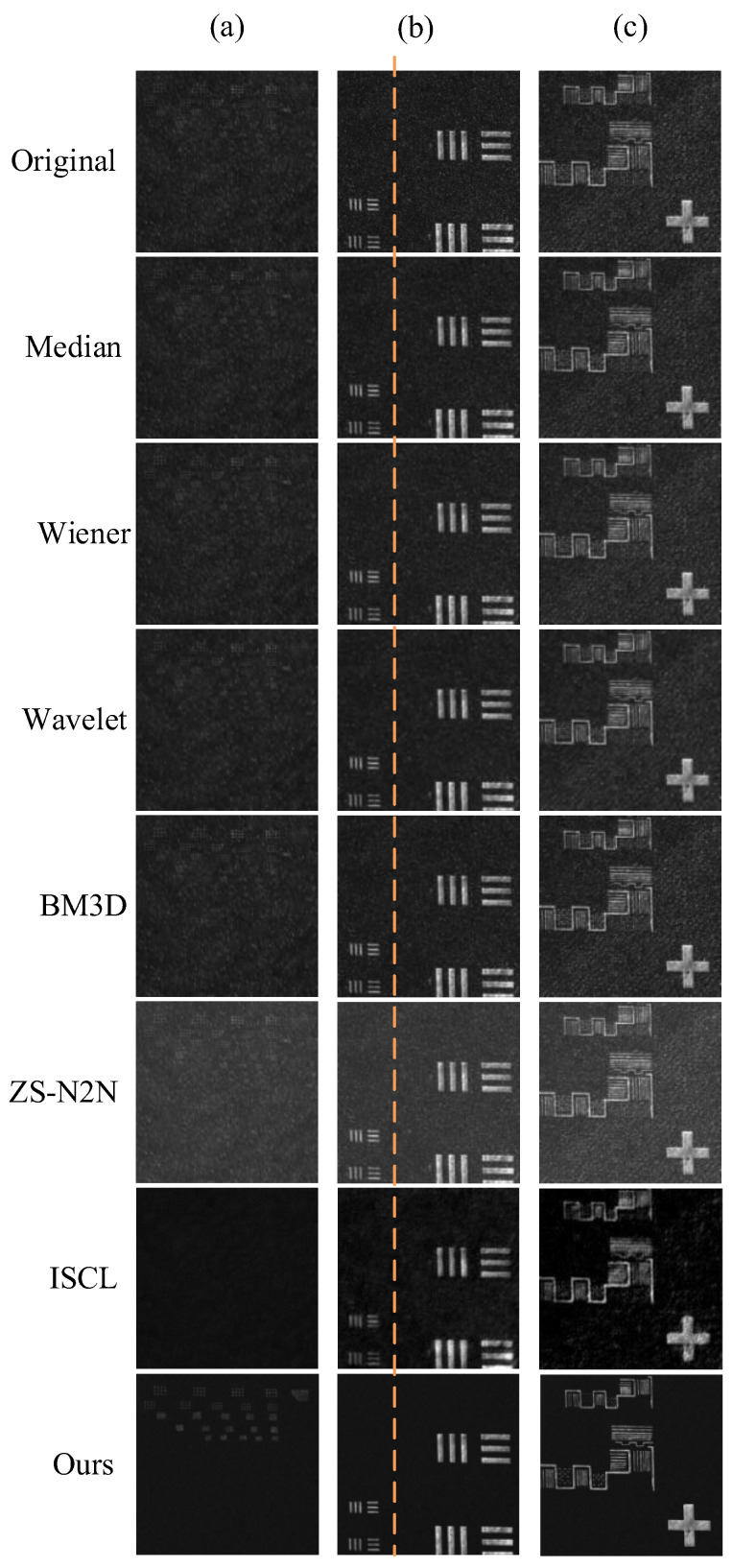
Comparison of denoising results for speckle noise images using different methods. (**a**–**c**) represent three typical images. The orange line denotes the region along which intensity values are extracted for uniformity evaluation.

As shown in Figure 7, the denoising performance of different methods is compared. Traditional image processing methods have limited effectiveness in suppressing speckle noise and cannot effectively remove a large amount of noise. Adjusting parameter settings can produce varying denoising effects, but as the filter size increases, the noise suppression improves while causing image blurring. ZS-N2N performs well in general denoising tasks, but due to its relatively simple network structure, it performs poorly in suppressing speckle noise and causes undesirable enhancement of overall image brightness. ISCL, as another unsupervised method based on CycleGAN, shows relatively strong performance. Ultimately, the proposed method in this paper demonstrates superior denoising effects, successfully removing noise while preserving image details and overall brightness.

**Figure 8:**
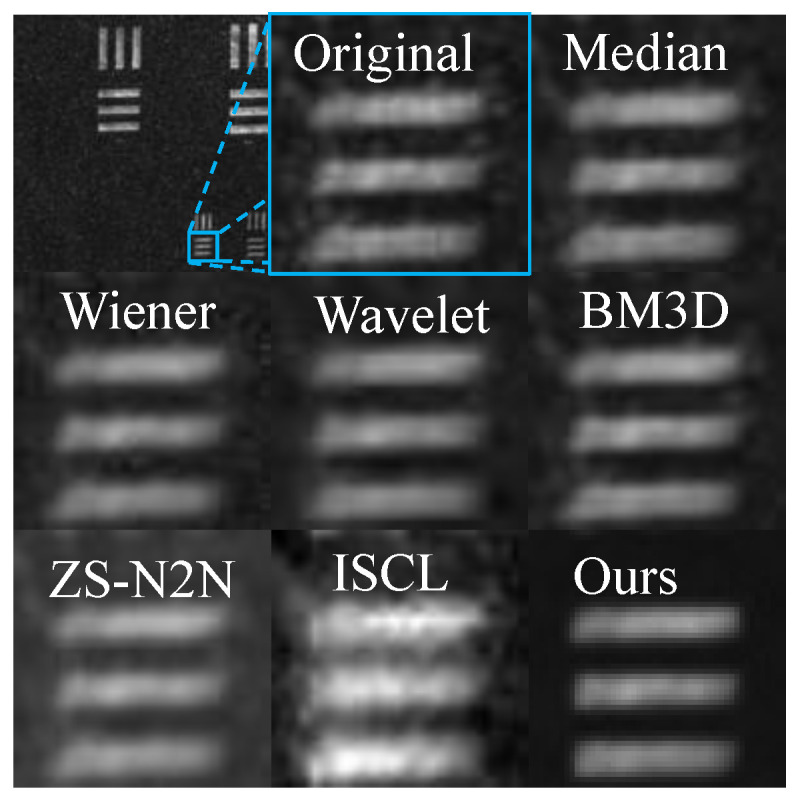
Visual comparison of denoising results on a resolution test sample. The top-left image shows the full field of view, with a marked region of interest. Each subsequent sub-image shows a 50 × 50-pixel patch cropped from the same region and displayed at its original resolution without interpolation. Our method preserves edge integrity while effectively suppressing speckle noise.

By zooming in on the images, the details of the denoised images using different methods in Figure 8 can be observed more clearly. Traditional filtering methods not only blur the overall image but also introduce undesirable artifacts, such as the blocky structures produced by Wiener filtering, which degrade the image quality and are not ideal. In contrast, the proposed PCGAN method effectively preserves visual details, achieving superior denoising performance.

**Figure 9:**
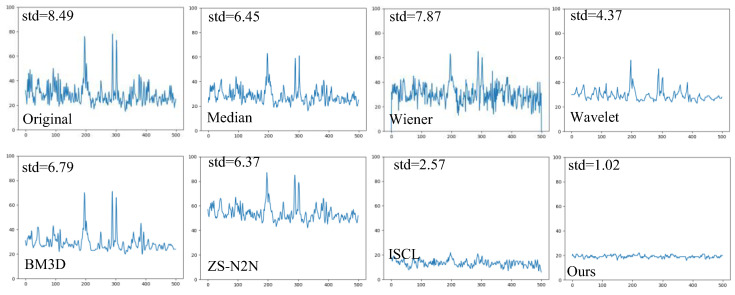
Line chart of the statistical distribution of the straight line of grayscale in Figure 8.

Additionally, we performed a statistical analysis of the pixel intensity values along a line containing only the background in the images from Figure 7, and the results are plotted as a line graph in Figure 9. The flatter the line, the better the denoising effect. In the original image, significant fluctuations in the adjacent pixel intensity values caused a large overall variation in the line graph. To measure this fluctuation, we calculated the standard deviation of the pixel intensities along the line. Among all the methods, our method produced the flattest line with the lowest standard deviation, indicating the best denoising performance, and the standard deviation can serve as a reliable quantitative evaluation metric.

The quantitative metrics were averaged over 500 independently collected and registered image pairs, covering diverse sample regions and speckle realizations. The quantitative results presented in Table 5 highlight the significant differences between various methods in noise suppression and detail preservation. Traditional techniques, such as median filtering, Wiener filtering, wavelet transform, and BM3D, perform moderately in noise suppression but have significant limitations in preserving image details. For example, median filtering and Wiener filtering degrade details as the window size increases, while the C-value and MSE decrease accordingly. Wavelet transform shows consistent performance but with moderate results, while BM3D performs excellently under low noise conditions, but its performance worsens with increased noise levels, leading to a decrease in SNR and SSIM values.

In contrast, advanced methods like ISCL and our method demonstrate markedly superior performance. Among them, the proposed method stands out, excelling in all evaluation metrics. It not only achieves strong noise suppression through low C-values but also excels in detail preservation with high SNR values. Furthermore, the proposed method maintains excellent overall image quality, as reflected in its optimal C-value. Compared to traditional methods, the proposed approach consistently outperforms them in noise reduction, detail retention, and overall image quality, making it especially suitable for image processing applications that require strict detail preservation and effective noise removal.

## 4. Conclusions

This paper presents a novel speckle noise suppression method for laser-based imaging that integrates physical constraints into an unsupervised learning framework. By incorporating both the statistical properties of light field perturbations and the image gradient consistency as guidance terms, the proposed method enables effective speckle suppression without requiring paired clean–noisy datasets.

Designed for coherent ultraviolet laser microscopy, the method demonstrates excellent denoising performance while preserving fine structural details and significantly improving the signal-to-noise ratio (SNR), a critical factor in high-resolution microscopic imaging. Although the approach involves calculating gradient-based constraints during training, this does not affect inference speed. Once trained, the model performs denoising in real time, making it suitable for deployment in practical and industrial settings.

While this study focuses on UV microscopy, the underlying physics-informed framework holds potential for extension to other coherent laser-based imaging modalities, such as optical coherence tomography (OCT) and digital holography. With appropriate adaptation to modality-specific characteristics, the proposed approach could address speckle-related image degradation more broadly in the future.

## Figures and Tables

**Table 1:** Speckle suppression results of different generators in the test set.

	Encoder and Decoder	U-Net	U-Net + EESP
MSE	10.45	7.54	6.98
SSIM	0.92	0.91	0.95
C	0.56	0.35	0.34
SNR	5.23	7.12	7.81

**Table 2:** Speckle suppression effects under different KL loss weights.

$\lambda_{1}$	0	0.1	0.2	0.5	0.8	1	2	5
MSE	7.83	7.81	7.42	7.22	7.12	6.98	9.89	12.56
SSIM	0.90	0.90	0.91	0.91	0.92	0.95	0.88	0.66
C	0.54	0.54	0.55	0.39	0.38	0.34	0.32	0.31
SNR	6.81	6.82	6.78	6.99	7.71	7.81	5.43	4.56

**Table 3:** Results of speckle suppression with different gradient loss weights.

$\lambda_{2}$	0	0.1	0.2	0.5	0.8	1	2	5
MSE	7.73	7.31	7.31	7.12	6.89	6.98	7.12	8.54
SSIM	0.85	0.88	0.91	0.93	0.95	0.95	0.91	0.81
C	0.44	0.43	0.44	0.35	0.32	0.34	0.34	0.35
SNR	6.87	6.88	6.92	7.76	7.82	7.81	6.52	6.12

**Table 4:** Ablation experiment results for the three loss functions. √ indicates that in the experiment, we employed this loss function.

KL Loss	Gradient Loss	MSE	SSIM	C	SNR
√		6.92	0.88	0.41	7.13
	√	6.95	0.86	0.44	7.05
		7.21	0.82	0.45	6.56
√	√	6.79	0.95	0.31	7.92

**Table 5:** Quantitative results of different methods.

Methods	Parameter	MSE	SSIM	C	SNR
Original	Null	9.55	0.78	0.53	5.54
Median	W: 3 × 3;	8.23	0.65	0.58	6.32
	W: 5 × 5;	8.12	0.63	0.56	6.44
	W: 7 × 7;	7.78	0.62	0.54	6.54
Wiener	W: 3 × 3;	8.89	0.72	0.48	6.78
	W: 5 × 5;	8.45	0.71	0.47	6.98
	W: 7 × 7;	8.12	0.67	0.46	7.54
Wavelet	M: “db2”, DS: 3;	9.56	0.83	0.47	6.82
	M: “db3”, DS: 3	9.12	0.81	0.48	7.06
BM3D	σ: 9;	8.54	0.88	0.51	7.32
	σ: 16;	8.82	0.85	0.49	7.41
	σ: 25	8.23	0.81	0.47	7.59
ZS-N2N	Same Training Set	7.34	0.92	0.38	7.51
ISCL	Same Training Set	7.12	0.91	0.35	6.98
Ours	Same Training Set	**6.79**	**0.95**	**0.31**	**7.92**

## Data Availability

Data underlying the results presented in this paper are not publicly available at this time but may be obtained from the authors upon reasonable request.
